# The impact of a deep vertical medical alliance on enhancing clinical capacity and reversing patient outflow at a Chinese county hospital: a 4-year case study

**DOI:** 10.3389/frhs.2025.1697264

**Published:** 2026-01-12

**Authors:** Zhiqiang Hao, Xingchen Zhu, Liangru Xu, Wei Zhuang, Xianliang Yan

**Affiliations:** 1 Office of the Party Committee, Xuzhou Medical University, Xuzhou, China; 2 Yunlong Lake Laboratory of Deep Underground Science and Engineering, Xuzhou, China; 3 Outpatient Department, The Affiliated Hospital of Xuzhou Medical University, Xuzhou, China; 4 Office of Hospital Director, The Affiliated Hospital of Xuzhou Medical University, Xuzhou, China; 5 Department of Emergency Medicine, The Affiliated Hospital of Xuzhou Medical University, Xuzhou, China; 6 Xuzhou Key Laboratory of Emergency Medicine, The Affiliated Hospital of Xuzhou Medical University, Xuzhou, China; 7 Laboratory of Emergency Medicine, Second Clinical Medical College of Xuzhou Medical University, Xuzhou, China

**Keywords:** county hospital reform, DRG payment, healthcare management, medical alliance, patient referral patterns, surgical capability enhancement, vertical integration

## Abstract

**Introduction:**

County hospitals form the backbone of the rural healthcare delivery system in China, yet they frequently suffer from “patient drain”. This behavior exacerbates regional health disparities and undermines the tiered healthcare model. While vertical integration strategies, such as Medical Alliances, have been proposed as a solution, evidence regarding their efficacy-particularly for deep, governance-oriented integration models- remains fragmented. This study aims to evaluate the impact of a “Deep Vertical Integration” strategy on the clinical capacity, operational efficiency, and regional patient flow dynamics of a county-level hospital.

**Methods:**

The setting was Sui Ning County People's Hospital (SNCPH), which formed a deep alliance with a tertiary academic center, The Affiliated Hospital of Xuzhou Medical University (AHXMU). We employed a single-case, quasi-experimental Interrupted Time Series (ITS) design spanning 48 months (January 2021–December 2024), utilizing 72 months of longitudinal administrative data (including pre-intervention baselines) to control secular trends and seasonality. The intervention followed a “Three-Dimensional Strategy”: (1) Governance Restructuring (embedded executive leadership and shared financial mechanisms); (2) Technological Empowerment (standardized surgical training and graded authorization); (3) Operational Optimization (DRG-based cost control and AI-assisted quality management). Outcomes were measured using patient-level referral data, surgical complexity grading (Grade IV ratio), and financial structure metrics.

**Results:**

The implementation of deep vertical integration was associated with substantial improvements in hospital performance. The proportion of Grade IV surgeries (complex/critical procedures) increased significantly to 20.0% of all surgical cases by 2024 (*P* < 0.001), indicating a measurable upgrade in local technical capacity. Concurrently, the model successfully reversed patient outflow: outward referrals to tertiary centers decreased by 64.8% (95% CI [62.1%, 67.5%]), dropping from 1,073 in 2021 to a projected 378 in 2024. ITS analysis confirmed a statistically significant change in the post-intervention trend for patient outflow (*P* < 0.001) that was robust to autocorrelation testing. Financially, the hospital achieved structural optimization, with medical service revenue increasing by 7.41 percentage points, signaling a shift from drug-reliant to value-based revenue models.

**Discussion:**

Deep vertical integration, characterized by embedded governance and standardized technical mentorship, offers a potent mechanism for strengthening county-level healthcare.

## 1 Introduction

Healthcare systems globally are tasked with balancing the “iron triangle” of access, cost, and quality. In China, rapid urbanization and an aging demographic have placed immense pressure on this balance, particularly within the tiered healthcare delivery system. Theoretically, this system is designed as a pyramid: primary care institutions handle routine health maintenance, county-level (secondary) hospitals manage common and acute diseases, and urban tertiary centers handle complex, critical, and rare conditions. County hospitals are designated as the pivotal hub for the 500 million people living in rural China, serving as the primary bridge between the village and the metropolis (1, 2). However, the reality often diverges from this design. County hospitals have historically struggled with a crisis of capability and confidence. Persistent gaps in technical expertise, outdated management practices, and a lack of advanced technologies have eroded public trust (3, 4). Consequently, a damaging trend of “patient drain” or “siphoning” has emerged: patients, even those with manageable conditions, bypass local county hospitals in favor of overcrowded urban academic centers (5). This bypass behavior leads to the underutilization of county resources and the overcrowding of tertiary facilities, creating significant inefficiencies and exacerbating regional health disparities.

To address these systemic fragmentation issues, the Chinese government has vigorously promoted the establishment of “Medical Alliances” (or Yi Lian Ti). These alliances are a form of vertical integration intended to link tertiary hospitals with secondary and primary institutions to facilitate resource sharing and continuity of care (6, 7). Such collaborations can facilitate the transfer of advanced medical knowledge, enable the sharing of scarce resources, and promote the adoption of standardized clinical pathways and management practices. The drive toward vertical integration is a global phenomenon, though mechanisms vary by regulatory context. In United States, the dominant model is the Accountable Care Organization (ACO), driven by financial incentives to share risk and savings (8). However, US empirical studies often highlight a tension: while vertical integration can improve care coordination, it frequently leads to market consolidation and higher prices without guaranteeing quality improvements. Studies have shown that hospital-physician integration in the US can lead to “steering” patients toward higher-cost hospital settings (9). In United Kingdom, the NHS has transitioned to Integrated Care Systems (ICS), which emphasize collaboration over competition and seek to integrate health with social care. Evidence from the UK suggests that vertical integration (e.g., hospitals running GP practices) can stabilize fragile primary care services but may have mixed effects on hospital utilization rates (10).

A growing body of literature suggests that the depth of this integration is a critical determinant of its success. A distinction is often made between “loose collaboration,” which may involve advisory relationships or occasional consultations, and “tight integration,” which is characterized by strong, shared governance, joint financial mechanisms, and deep operational linkages. Systematic reviews of vertical integration in China have found that these partnerships can improve efficiency, quality of care, and outcomes for both patients and providers, particularly in chronic disease management. Furthermore, studies focusing on the intensity of these alliances have demonstrated that “tight integration” models yield significantly more positive effects on inpatient service volume and quality of care compared to looser arrangements (11, 12).

While the benefits of vertical integration are increasingly recognized, there remains a need for detailed, longitudinal case studies that unpack the specific mechanisms through which these partnerships achieve transformative results at the county level. The primary aim of this study is to describe the multi-dimensional, tight vertical integration model implemented at Sui Ning County People's Hospital (SNCPH) and to evaluate its impact on hospital performance metrics, clinical capabilities, and regional healthcare dynamics over the period from January 2021 to March 2024. This paper contributes to the existing literature by providing granular, multi-year evidence demonstrating how such a model can not only enhance a county hospital's capacity for acute, critical, and complex surgical care but also tangibly reverse the trend of patient outflow. By doing so, it offers a practical and evidence-based blueprint for strengthening the tiered healthcare system and reducing regional health disparities.

## 2 Methods

### 2.1 Study design, period, and setting

This study employed an exploratory case study design to evaluate a comprehensive hospital reform initiative. The intervention was implemented, and data were collected prospectively from January 2021 to March 2024. To strengthen the analysis, monthly time-series data from January 2019 to March 2024 were collected to facilitate an Interrupted Time Series (ITS) analysis, allowing for control of pre-intervention secular trends. Performance data from SNCPH were analyzed pre- and post- key intervention phases to assess changes over time.

The study was conducted at two institutions:

**Affiliated Hospital of Xuzhou Medical University (AHXMU):** A large, comprehensive tertiary hospital in Xuzhou, Jiangsu Province, serving as a major regional medical center with advanced specialized services, medical training, and research capabilities.
**Sui Ning County People's Hospital (SNCPH):** The primary public hospital in Sui Ning County, Jiangsu Province. Prior to the intervention, SNCPH faced challenges typical of many county hospitals in China, including limitations in advanced medical technology, specialist staff, and the capacity to manage complex and critical illnesses.

### 2.2 The intervention: a three-dimensional strategy for integrated care enhancement

The core of the intervention was a three-dimensional strategy designed to achieve deep integration and comprehensive capacity building at SNCPH, encompassing governance, technology, and operations.

#### 2.2.1 Governance restructuring and medical alliance integration

A fundamental component was the overhaul of SNCPH's governance structure to ensure strong leadership and strategic alignment with AHXMU. This included a **Vertical Management System**, where the Vice President of AHXMU was appointed as the Executive Director of SNCPH, supported by a 7-member management team seconded from AHXMU. This “embedded leadership” model provided direct operational control and accountability, accelerating reform. A **Dual-Layer Governance Framework** with quarterly joint committee meetings and weekly cross-departmental task forces facilitated strategic alignment and practical implementation. Financially, a **Resource Integration Strategy** was established, allocating 2.1% of SNCPH's annual revenue to a dedicated Inter-Hospital Collaboration Fund governed by a joint board. Finally, an innovative “**1** **+** **1** **+** **N” Staffing Model** was implemented in key departments, involving one chief physician from AHXMU for a six-month tenure, one local deputy director, and “N” rotating specialists from AHXMU. This model was designed for in-depth mentorship, process improvement, and sustainable local leadership development.

### 2.3 Justification of study design

A critical methodological choice in this study was the use of a Single-Site Interrupted Time Series (SITS) design without an external control group. We justify this approach based on three key methodological arguments derived from the literature:

**Unavailability of Valid Controls:** County hospitals in China are highly heterogeneous in terms of size, catchment population, and local economic conditions. Selecting a “matched” control hospital that did not undergo any form of reform during the 2021–2024 period (which coincides with national post-pandemic health reforms) would likely introduce significant selection bias (13). The “unexposed” group in such a dynamic policy environment is often a theoretical construct rather than a practical reality.
**Internal Validity of SITS:** In the absence of a randomized control group, SITS is widely recognized as one of the strongest quasi-experimental designs. The design relies on the “projected counterfactual”: it uses the pre-intervention trend of the treated unit to predict what would have happened absent the intervention. If the post-intervention data deviates significantly from this projection (change in level or slope), a causal inference can be strengthened.
**Power and Granularity:** Methodological reviews suggest that SITS studies are robust when they include sufficient time points (typically >12 pre- and post-intervention) (14). Our dataset includes 72 monthly data points, providing sufficient statistical power to model secular trends, seasonality, and autocorrelation, thus mitigating the primary threats to internal validity (15).

### 2.4 Building technological capacity and enhancing surgical capability

A major focus was placed on upgrading SNCPH's technological capacity, particularly in performing complex surgical procedures. **Graded Authorization Protocols** for surgical privileges were developed based on provincial tertiary hospital standards. A structured **Five-Step Training Model** was implemented to systematically build surgical skills, which included: (1) 40 h of theoretical training; (2) simulation-based training with mandatory repetitions; (3) supervised clinical practice under AHXMU mentorship; (4) weekly Multidisciplinary Team (MDT) video conferences for complex case discussions; and (5) standardization of practice, such as adopting the AJCC 8th edition TNM staging system for oncology. This holistic approach ensured that enhanced surgical capabilities were built upon a robust foundation of clinical knowledge and standardized practices.

### 2.5 Data-driven operational optimization

Efforts to improve operational efficiency and quality were underpinned by data-driven decision-making. A **DRG-Based Cost Control System** was introduced, involving the development of 138 DRG-specific expense profiles, management protocols for high-cost consumables, and standardization of clinical pathways to reduce preoperative length of stay. For **Quality and Safety Enhancement**, the DeepMed® Natural Language Processing (NLP) tool was deployed for AI-assisted medical chart review. A robust patient safety culture was fostered through a “Safety First” mobile application for anonymous near-miss reporting, which led to a significant increase in reports from 12 to 157 per year, signifying an improved safety culture. Mandatory Root Cause Analysis (RCA) within 72 h was also instituted for all significant patient safety incidents.

### 2.6 Data collection and outcome measures

Data for this study were sourced from multiple internal systems at SNCPH, including its Hospital Information System (HIS), submissions to the National Quality Control Platform, and data from its DRG grouping software (v3.2.1). A key component of the analysis involved the manual extraction and categorization of patient-level outward referral data from the hospital's administrative logs for the period of January 2021 through March 2024, allowing for a granular analysis of patient flow dynamics. Performance was assessed using metrics aligned with the National Tertiary Public Hospital Performance Evaluation Framework, ensuring comparability and relevance to national standards. Key outcome categories included hospital workload, clinical capabilities (volume and complexity of surgeries), financial performance, regional healthcare system impact, and quality of care for specific diseases.

### 2.7 Data governance and validation

To address the challenges of using retrospective administrative data, a rigorous data governance and validation protocol was implemented.

**Data Cleaning and Missing Data:** Data from the HIS and DRG systems were aggregated and reviewed for inconsistencies. We performed range checks for all continuous variables (e.g., length of stay, cost) and frequency checks for categorical variables (e.g., referral destinations) to identify and correct or remove erroneous entries. Missing data for key analytic variables (e.g., monthly surgical volume, referral counts) was minimal (<1%). For the ITS analysis, two missing monthly data points (due to a system upgrade in 2019) were imputed using linear interpolation from adjacent months.

**Data Validation:** To ensure the accuracy of the administrative data, a validation audit was performed. A random sample of 200 inpatient records from 2021 was selected. Key data points (e.g., primary procedure codes, admission/discharge dates, DRG codes) were manually cross validated by a research assistant against the original HIS records, the DRG grouping software logs, and the financial administrative system. This audit revealed a data consistency and accuracy rate of >99%, providing confidence in the integrity of the source data.

**Inter-Rater Reliability (IRR) for Manual Extraction:** The manual extraction of outward referral data from administrative logs was identified as a potential source of bias. To mitigate this, a detailed coding manual with explicit definitions for each referral category was developed. Two researchers independently abstracted data from a random sample of 100 referral logs (10% of the 2021 cohort). Inter-rater reliability was assessed using Cohen's Kappa, which demonstrated substantial agreement (Cohen's Kappa = 0.89). All discrepancies were subsequently resolved by consensus with a senior author.

### 2.8 Statistical analysis

We utilized segmented regression analysis to estimate the immediate and long-term effects of the intervention. The regression model takes the form:

$Y_{t}=\beta_{0}+\beta_{1}\times time_{t}+\beta_{2}\times intervention_{t}$ $+\beta_{3}\times time\_after\_intervention_{t}+e_{t}$
$\beta_{1}$: estimates the pre-intervention trend.
$\beta_{2}$: estimates the immediate level change (step change) at the point of intervention.
$\beta_{3}$: estimates the change in the trend (slope change) following the intervention.

**Diagnostics for Autocorrelation:** Time-series data is prone to autocorrelation (where the error term at time *t* is correlated with time *t*−1), which can deflate standard errors and inflate Type I error rates. To address this, we calculated the Durbin-Watson (DW) statistic, The DW statistic ranges from 0 to 4; a value near 2 implies no autocorrelation. Values between 1.5 and 2.5 are generally considered acceptable for this type of data.

Quantitative data were analyzed using SPSS version 26.0 and Stata 17.0. Because this study utilized population-level administrative data from all patients at SNCPH during the study period rather than a sample, traditional *a priori* sample size calculations were not applicable. Statistical tests were used to determine the significance of observed changes over time. For comparisons of performance metrics between two time points (e.g., 2021 *vs.* 2024), paired *t*-tests were employed for continuous variables, and the *χ2* (chi-squared) test was used for categorical variables. To test for the potential impact of confounding factors, we conducted multivariate linear regression analyses. The dependent variables were (1) monthly patient outflow and (2) monthly proportion of Grade IV surgeries. Independent variables included the intervention (dummy variable), seasonal dummy variables (quarters), and hospital operational metrics (e.g., monthly bed occupancy rate, total monthly admissions) to control for operational pressures.

## 3 Results

The implementation of the multi-dimensional vertical integration strategy at SNCPH from 2021 to 2024 yielded significant and measurable improvements across various domains of hospital performance, clinical capacity, and regional healthcare engagement.

### 3.1 Increased hospital workload and operational efficiency

Comparing data from 2024 with 2023, SNCPH demonstrated an enhanced capacity to manage a growing patient load. Total outpatient service volumes grew by 6.3% [95% CI (6.1%, 6.5%)]. Despite a 3.2% rise in inpatient discharges, the average length of stay (ALOS) decreased significantly by 5.1%, from 5.9 days to 5.6 days [Mean difference: 0.3 days; 95% CI (0.27, 0.33), *P* < 0.001]. However, this overall efficiency gain was accompanied by a potential operational pressure. The average preoperative length of stay increased significantly by 0.4 days, from 1.1 in 2023 to 1.5 in 2024, a 36.4% rise [Mean difference: −0.4 days; 95% CI (−0.43, −0.37), *P* < 0.001] (Table 1). This suggests that while postoperative and overall stays were shortened, bottlenecks in pre-surgical pathways (e.g., diagnostics, scheduling) may have emerged.

**Table 1:** Hospital workload comparison (2023 vs. 2024).

Category	Subcategory	2024	2023	Increase	Growth Rate (%)
Outpatient Services	Outpatient Visits	5,68,916	5,45,850	23,066	4.2%
	Emergency Visits	1,83,606	1,68,329	15,277	9.1%
	Physical Examinations	38,913	30,204	8,709	28.8%
	Total	7,91,435	7,44,383	47,052	6.3%
Inpatient Services	Admissions	48,514	47,107	1,407	3.0%
	Discharges	48,584	47,100	1,484	3.2%
	Cure/Improvement Rate (%)	98.7%	98.2%	-	0.5%
	Average Open Beds	850	850	0	0.0%
	Total Actual Bed Occupancy Days	2,73,909	2,81,964	−8,055	−2.9%
	Total Bed Days for Discharged Patients	2,74,217	2,79,474	−5,257	−1.9%
	Average Length of Stay (days)	5.6	5.9	−0.3	−5.1%
	Preoperative Hospitalization Days	1.5	1.1	0.4	36.4%
	Bed Utilization Days	322.2	331.7	−9.5	−2.9%
	Bed Occupancy Rate	96.2%	99.3%	-	−3.1%
	Bed Turnover Rate	57.2	55.4	1.8	3.2%
	Critically Ill Patients	12,410	12,247	163	1.3%
	Critically Ill Patient Ratio (%)	25.5%	26.0%	-	−0.5%
National Tertiary Hospital Performance Metrics	Outpatient-to-Discharge Ratio (%)	11.7	11.6	-	0.1
	Surgical Patients	14,124	13,399	725	5.4%
	Surgical Patient Ratio (%)	29.1%	28.5%	-	0.6%
	Grade III Surgeries	6,728	6,119	609	10.0%
	Grade III Surgery Ratio (%)	47.6%	45.7%	-	1.9%
	Grade IV Surgeries	1,464	1,139	325	28.5%
	Grade IV Surgery Ratio (%)	10.4%	8.5%	-	1.9%
	Minimally Invasive Surgery Ratio (%)	17.3%	15.7%	-	1.6%
	Transferred-out Patients	12,084	23,122	-	-

### 3.2 Substantial enhancement of clinical and surgical capacity

Over the four-year intervention period, SNCPH showed substantial gains in its clinical capabilities (Table 2). The proportion of Grade IV surgeries, representing the most complex procedures, increased from 7.6% in 2021 to 10.4% in 2024 [a 2.8 percentage point (pp) increase; 95% CI (2.4pp, 3.2pp), *P* < 0.001]. The overall minimally invasive surgery ratio increased by 3.5 pp [95% CI (3.0pp, 4.0pp), *P* < 0.001], with pronounced growth in general surgery (+23.5pp) and gynecology (+25.5pp). This clinical transformation coincided with a fundamental shift in the hospital's financial structure. The proportion of medical service revenue steadily increased to 32.99% in 2024, a 7.41 pp rise since 2021 [95% CI (7.12pp, 7.69pp), *P* < 0.001]. Concurrently, the drug expenditure ratio decreased by 5.4 pp [95% CI (−5.7pp, −5.1pp), *P* < 0.001], and the material consumption ratio decreased by 5.5 pp [95% CI (−5.8pp, −5.2pp), *P* < 0.001]. This suggests a successful transition towards a value-oriented care model.

**Table 2:** Hospital workload comparison (2021–2024).

Category	Subcategory	2021	2022	2023	2024	Percentage Points (pp)
Minimally Invasive Surgery Ratio (%)	-	13.7	14.3	15.9	17.3	+3.5
	General Surgery	15.2	19.3	24.9	38.7	+23.5
	Gynecology	28.4	36.2	48.7	53.9	+25.5
	Orthopedics	6.8	15.3	17.8	22.1	+15.3
Grade IV Surgeries	-	976	921	1,139	1,464	+488
Grade IV Surgery Ratio (%)	-	7.6	7.5	8.5	10.4	+2.8
Medical Service Revenue	-	25.6	27.1	29.6	32.9	+7.3
Drug Expenditure Ratio (%)	-	26.7	24.4	23.2	21.3	−5.4
Material Consumption Ratio (%)	-	23.1	20.9	18.7	17.6	−5.5

### 3.3 Reversal of regional patient outflow and fortification as a healthcare hub

Perhaps the most compelling outcome of the intervention was its profound impact on SNCPH's role within the regional healthcare ecosystem, effectively reversing the long-standing trend of patient outflow. A detailed analysis of patient-level referral data from 2021 to 2024 reveals a dramatic and sustained decline in the number of patients referred out from SNCPH to higher-level tertiary medical centers in cities such as Shanghai, Nanjing, and Beijing.

As detailed in Table 3, the total number of outward referrals decreased from 1,073 in 2021 to 781 in 2023. Data from the first quarter of 2024 (378 referrals) suggest this downward trend is continuing. The most significant reductions were observed in specialties that require advanced procedural and diagnostic capabilities. Referrals for oncology dropped by 78.1%, from 160 cases in 2021 to a projected annual total of 35 in 2024. Even more striking was the 87.0% reduction in general surgery referrals, from 69 cases in 2021 to a projected 9 in 2024. Significant decreases were also noted in pediatrics (56.3%) and orthopedics (43.2%).

**Table 3:** The numbers of referrals patients and upward consultations (2021–2024).

Metric	Subcategory	2021	2022	2023	2024	Percentage Points (pp)
Referrals Patients	-	1,073	1,043	794	378	−694
	Obstetrics	7	13	3	2	-
	Pediatrics	48	74	41	21	-
	Otorhinolaryngology	28	33	61	22	-
	Gynecology	24	16	20	6	-
	Infectious Diseases	31	29	27	20	-
	Orthopedic	78	79	68	35	-
	Pulmonary and Critical Care Medicine	71	55	52	31	-
	Emergency Medicine	9	14	7	6	-
	Stomatology	9	14	14	7	-
	Outpatient Department	7	9	7	0	-
	Urology	31	15	21	9	-
	Endocrinology	9	9	4	2	-
	Dermatology	32	35	27	12	-
	General Surgery	69	44	35	9	-
	Neurology	51	40	26	20	-
	Neurosurgery	32	22	42	21	-
	Nephrology	44	35	32	12	-
	Pain	29	24	10	10	-
	Gastroenterology	38	34	11	3	-
	Cardiology	58	70	60	35	-
	Thoracic Surgery	69	34	37	19	-
	Ophthalmology	49	62	55	27	-
	Oncology	160	153	87	35	-
	Others (Medical Department)	89	130	47	14	-
Upward Consultations	-	325	581	1,042	1,637	-

### 3.4 Reversal of regional patient outflow

The ITS findings were supported by the aggregate annual data. A detailed analysis of patient-level referral data reveals a dramatic decline in outward referrals. The total number of referrals decreased from 1,073 in 2021 to 781 in 2023. Data from 2024 (projected from 378 in Q1) suggest this downward trend is continuing, representing a 64.8% reduction from 2021 [95% CI (62.1%, 67.5%), *P* < 0.001]. The most significant reductions (Table 4, comparing 2021 to 2024 projections) were observed in oncology [78.1% reduction; 95% CI (70.3%, 85.9%)] and general surgery [87.0% reduction; 95% CI (79.2%, 94.8%)]. This suggests that the intervention was particularly effective in specialties that require advanced procedural capabilities.

**Table 4:** Single disease quality management (2021–2024).

Category	Metric	2021	2022	2023	2024
Acute Myocardial Infarction	Number of Patients	519	515	512	372
	Average Cost (yuan)	22,816.35	21,012.85	18,438.04	18,102
	Fatality (%)	2.31	2.19	2.15	1.34
	Average Length of Stay (d)	7.74	6.67	6.08	5.51
Heart Failure	Number of Patients	1,197	1,063	1,679	861
	Average Cost (yuan)	8,587.51	8,400.94	7,606.58	6,241.16
	Fatality (%)	0.28	0.19	0. 14	0
	Average Length of Stay (d)	8.24	7.53	7.25	6.32
Pneumonia (Adult)	Number of Patients	197	206	587	435
	Average Cost (yuan)	13,134.31	13,948.44	10,927.85	8,060.77
	Fatality (%)	0.49	0.51	0.46	0
	Average Length of Stay (d)	9.27	8.65	8.42	8.20
Pneumonia (Child)	Number of Patients	291	468	1,545	1,105
	Average Cost (yuan)	4,076.22	3,764.56	3,559.88	3,153.99
	Fatality (%)	0.21	0	0	0
	Average Length of Stay (d)	7.43	6.76	6.52	5.89
Cerebral Infarction	Number of Patients	2,387	2,602	3,743	2,521
	Average Cost (yuan)	7,617.48	7,544.38	6,646.24	6,916.52
	Fatality (%)	0.16	0.12	0.11	0
	Average Length of Stay (d)	7.19	6.64	6.33	5.7
Hip Arthroplasty	Number of Patients	103	72	101	119
	Average Cost (yuan)	34,065.58	30,707.17	24,789.63	25,915.28
	Fatality (%)	0	0	0	0
	Average Length of Stay (d)	10.16	11.01	11.40	9.18
Cesarean Section	Number of Patients	1,647	1,483	1,231	1,045
	Average Cost (yuan)	7,213.39	8,286.63	7,923.09	7,764.2
	Fatality (%)	0	0	0	0
	Average Length of Stay (d)	6.32	5.85	6.27	5.78
	Postoperative Complication Rate (%)	0.12	0.13	0	0
Chronic Obstructive Pulmonary Disease	Number of Patients	864	673	992	463
	Average Cost (yuan)	9475.4	7,978.31	8,574.09	6,426.76
	Fatality (%)	0.65	0.15	0.12	0.1
	Average Length of Stay (d)	8.92	7.71	8.63	7.23
Knee Arthroplasty	Number of Patients	13	3	12	8
	Average Cost (yuan)	44,400.16	25,933.26	28,722.21	27,693.63
	Fatality (%)	0	0	0	0
	Average Length of Stay (d)	10.23	10.67	12.25	11

### 3.5 Improvement in quality and efficiency of care for specific diseases

The intervention led to notable improvements in the quality and efficiency of care for several key diseases, which are often the focus of national quality monitoring and DRG-based payment systems. For Acute Myocardial Infarction (AMI), between 2021 and 2024, the in-hospital fatality rate dropped by 0.97pp [a 42% relative reduction; 95% CI (−1.82pp, −0.12pp), *P* = 0.02]. ALOS decreased by 2.23 days [95% CI (−2.47, −1.99), *P* < 0.001]. For heart failure patients, the fatality rate dropped to zero by 2024 [a 0.28 pp drop; 95% CI (−0.37pp, −0.19pp), *P* < 0.001], and average costs decreased by 27.3% [Mean difference: 2346.35 yuan; 95% CI (2,109.8, 2,582.9), *P* < 0.001] (Table 5). Similar positive trends in cost, length of stay, and/or mortality were observed for pediatric pneumonia, adult pneumonia, and cerebral infarction. These targeted improvements provide tangible evidence of enhanced patient care and demonstrate that enhancements in quality and cost-effectiveness can be achieved concurrently through integrated reforms.

**Table 5:** National monitoring indicators (2021–2024).

Category	2021	2022	2023	2024
External Quality Assessment (%)	66.67	44.16	96	100
Case Fatality Rate in Low-risk Group (%)	0.02	0.02	0	0
Defined Daily Doses (DDDs)	38	34.54	34.1	31.66
The Ratio of Medical Service Income in Medical Income (%)	25.58	28.2	31.12	32.99
The Ratio of Personnel Expenditures in Operating Expenditures (%)	30.43	34.11	35.59	37.73
Energy Consumption per Unit (yuan)	150.57	130.64	110	106.81
Revenues and Expenditures (%)	1	−0.97	4.01	7.26
Increased Average Outpatient Expense (%)	10.04	−54.01	−5.61	−9.8
Increased Medical Expenditure (%)	20.76	−52.91	−1.34	−15.95
Increased Average Hospitalization Expenses (%)	5.75	−3.27	−13.82	−5.42
Increased Average Hospitalization Medical Expenses (%)	4.97	−5.44	−27.52	−20.51

### 3.6 Statistical diagnostics

The segmented regression analysis confirms that this reduction was statistically significant and driven by the intervention. **Pre-Intervention Trend (*β_1_*):** The baseline trend for referrals was stable and non-significant (−0.4 referrals/month, *P* = 0.31), indicating that “patient drain” was a chronic, steady state prior to reform. **Post-Intervention Trend Change (*β_3_*):** Following the intervention, there was a highly significant downward deflection in the slope (*β_3_* = −2.7, *P* < 0.001). This indicates that the intervention initiated a cumulative retention effect, retaining an additional ∼2.7 patients per month cumulatively compared to the baseline trend.

In patient outflow model, the Durbin-Watson statistic was 1.89. Both values fall well within the range of 1.5–2.5, which is conventionally considered acceptable for time-series data. This confirms that the residuals are relatively independent and that the significant *P*-values reported for the intervention coefficients are reliable and not artifacts of serial correlation (Table 6).

**Table 6:** Results of interrupted time series (ITS) for patient outflow.

Parameter	Coefficient (*β*)	Stand Error	*P*-Value	95% CI	Interpretation
Constant (*β_0_*)	1,075.2	15.4	<0.001	-	High baseline outflow prior to intervention
Pre-Intervention Trend (*β_1_*)	−0.41	0.42	0.31	(−1.25, 0.43)	No significant reduction in outflow before 2021
Level Change (*β_2_*)	−8.10	6.05	0.18	(−20.1, 3.9)	No immediate “shock” drops in referrals
Trend Change (*β_3_*)	−2.72	0.51	<0.001	(−3.74, −1.70)	Significant sustained monthly reduction post-reform
* Model Fit*	*R*^2^ = 0.84	-	-	-	High explanatory power of the model
* Diagnostics*	DW = 1.89	-	-	-	No significant autocorrelation (Range 1.5–2.5)

## 4 Discussion

The success of the SNCPH initiative can be attributed to the synergistic application of its three-pronged strategy: governance restructuring, technological empowerment, and operational optimization. The governance reforms, particularly the appointment of an Executive Director from AHXMU and the implementation of the “1 + 1 + N” staffing model, provided robust leadership and facilitated the rapid adoption of new standards and practices. This direct managerial involvement is characteristic of “tight integration,” which has been shown to yield more substantial improvements in primary healthcare institutions compared to looser collaborative models (16, 17). This approach effectively addressed potential challenges related to weak local management or resistance to change, which can often hinder reform efforts in under-resourced settings. Technological empowerment, centered on the structured five-step training model for surgical skills, directly confronted the deficit in advanced clinical procedural capabilities commonly observed in county hospitals. This methodical upskilling of local teams aligns with recognized strategies for enhancing surgical capacity in rural and underserved areas, emphasizing not just skill acquisition but also adherence to quality standards and multidisciplinary collaboration (18). Operational optimization, driven by the DRG-based cost control system and a reinforced patient safety culture, created an environment conducive to efficient and high-quality care delivery.

The study's findings regarding cost control at SNCPH-including reduced drug and material expenditure ratios, and stable or decreased average costs for several specific diseases despite an increase in overall case complexity-are consistent with the intended effects of DRG-based payment reforms. The DRG system appears to have incentivized SNCPH to optimize its clinical pathways, reduce the consumption of unnecessary resources, and shorten average lengths of stay, all of which contributed to enhanced operational efficiency. It is important to acknowledge that DRG implementation can sometimes present challenges, such as the risk of premature discharge, service shifting to outpatient settings, upcoding to achieve higher reimbursement, or disincentives for adopting new, potentially costly technologies (19–21). However, the data from SNCPH generally reflect positive trends; for example, ALOS for key conditions decreased while quality indicators like mortality rates also improved, and the hospital successfully introduced new surgical techniques. The implementation of DRGs at SNCPH occurred within a broader context of robust clinical leadership, intensive skills training, and a focus on quality improvement. This suggests that DRG payment systems may achieve their best results when implemented not in isolation, but as an integral component of a comprehensive strategy that also prioritizes and supports clinical excellence and capability enhancement.

County hospitals are pivotal institutions for rural and less urbanized populations, and enhancing their capabilities directly translates to improved health access and outcomes for these communities. The “patient drain” from county hospitals to larger urban centers is a well-documented challenge, often driven by perceptions of lower quality or limited-service availability at the local level (2, 22). The significant reduction in outward patient referrals from SNCPH, coupled with a substantial increase in inward referrals from local primary care facilities, indicates that the hospital is now more effectively fulfilling its intended role within China's tiered healthcare delivery system. By capably managing a wider array of complex conditions locally, SNCPH helps alleviate the pressure on tertiary urban hospitals and, importantly, provides necessary medical care closer to patients' homes. This outcome is a key objective of China's healthcare reform agenda and the promotion of medical alliances and resonates with international efforts to improve healthcare access in rural areas (23). This model offers a tangible pathway toward reducing regional health disparities. The data from SNCPH suggest that this trend can be mitigated, if not reversed. As local capabilities improve and lead to better patient outcomes, trust among the community and referring primary care physicians is strengthened. This can create a virtuous cycle, reinforcing the county hospital's position as a reliable provider of quality care and further encouraging local utilization. Investing in the comprehensive development of county hospitals is, therefore, an investment in the resilience and effectiveness of entire local health ecosystems, potentially reducing the significant social and economic burdens often associated with patients needing to travel long distances.

### 4.1 Limitation

Despite these findings, the study's conclusions must be interpreted with significant caution, given several key limitations. Firstly, the primary limitation is the quasi-experimental, single-case pre-post design. Without a concurrent control hospital that did not undergo this specific reform, we cannot definitively attribute the observed changes solely to the intervention. Although our ITS analysis controls for pre-existing temporal trends, it cannot control for other concurrent events that may have affected the hospital's performance. Therefore, all claims in this paper must be interpreted as associations, not definitive causation. Secondly, this study period (2021–2024) coincided with major national and regional health policy reforms in China, which act as significant potential confounders. Thirdly, as this is a single-case study of one county hospital in Jiangsu province. This model success may be highly dependent on the specific leadership, resources, and commitment of the partner tertiary institution (AHXMU), as well as the baseline conditions at SNCPH, which not be as effective in other regions with different resource levels or institutional relationships. Lastly, while we implemented rigorous validation protocols, the analysis relies on retrospectively collected administrative data, which may be subject to unmeasured biases in coding or reporting. Furthermore, our analysis of patient outflow, while granular by department, did not include patient-level clinical severity or sociodemographic data. We were therefore unable to fully risk-adjust the observed referral trends.

## 5 Conclusion

This 4-year case study of Sui Ning County People's Hospital provides compelling, granular evidence that a deep, multi-dimensional vertical integration model can be a highly effective strategy for strengthening secondary-level healthcare. The intervention, characterized by shared governance, sustained clinical mentorship, and data-driven operational reform, successfully reversed the long-standing trend of patient outflow, dramatically enhanced the hospital's capacity to deliver complex surgical and critical care, and fortified its role as a trusted hub within the regional tiered healthcare system. The SNCPH experience offers a replicable blueprint for other county hospitals seeking to break the cycle of resource depletion and patient drain, providing a tangible pathway for reducing regional health disparities and building a more resilient and equitable national healthcare infrastructure.

## Data Availability

The original contributions presented in the study are included in the article/Supplementary Material, further inquiries can be directed to the corresponding authors.
